# Similar Odor Discrimination Behavior in Head-Restrained and Freely Moving Mice

**DOI:** 10.1371/journal.pone.0051789

**Published:** 2012-12-18

**Authors:** Nixon M. Abraham, Delphine Guerin, Khaleel Bhaukaurally, Alan Carleton

**Affiliations:** 1 Department of Basic Neurosciences, School of Medicine, University of Geneva, Geneva, Switzerland; 2 Geneva Neuroscience Center, University of Geneva, Geneva, Switzerland; Université Lyon, France

## Abstract

A major challenge in neuroscience is relating neuronal activity to animal behavior. In olfaction limited techniques are available for these correlation studies in freely moving animals. To solve this problem, we developed an olfactory behavioral assay in head-restrained mice where we can monitor behavioral responses with high temporal precision. Mice were trained on a go/no-go operant conditioning paradigm to discriminate simple monomolecular odorants, as well as complex odorants such as binary mixtures of monomolecular odorants or natural odorants. Mice learned to discriminate both simple and complex odors in a few hundred trials with high accuracy. We then compared the discrimination performance of head-restrained mice to the performance observed in freely moving mice. Discrimination accuracies were comparable in both behavioral paradigms. In addition, discrimination times were measured while the animals performed well. In both tasks, mice discriminated simple odors in a few hundred milliseconds and took additional time to discriminate the complex mixtures. In conclusion, mice showed similar and efficient discrimination behavior while head-restrained compared with freely moving mice. Therefore, the head-restrained paradigm offers a relevant approach to monitor neuronal activity while animals are actively engaged in olfactory discrimination behaviors.

## Introduction

Highly precise behavioral paradigms [1], [2], [3], [4], [5], and recent technical advancements in the cell type specific modification of neuronal circuitry [11], [12], [13], [14], [15] now bring us the challenging task of monitoring neuronal activity while animals are actively involved in specific behaviors over long periods of time. The most important parameters in investigating the neuronal basis of a specific behavior are, (1) establishing behavioral readouts with reproducibility over hundreds of trials, (2) recording the behavioral readouts with the temporal resolution of neural events. To facilitate the recording of neural events from an actively behaving animal on a single cell basis or population basis (reviewed in [16]), the best strategy is to keep the animals head-restrained [10], [17], [18]. But whether behavioral readouts from a head-restrained animal can be compared with that of a freely moving animal still remains unanswered.

Although a head-restrained strategy optimizes the requirements for dissecting neuronal basis of specific behaviors, limited attempts have been made for detailed analysis of olfactory specific behaviors under head-restrained conditions [19], [20], [21]. We, therefore, established a robust olfactory behavioral paradigm in head-restrained mice using a go/no-go operant conditioning paradigm that has previously been used for freely moving animals [1], [22]. Psychophysical reaction time measurements provide the time limit within which the neural events underlying a specific behavior happen [23]. For olfactory tasks, in freely moving subjects, we define discrimination times (DTs) as comparison of reaction times computed when animals are discriminating stimuli at high accuracies [1], [11]. DTs have already been shown as one of the most sensitive parameters to study the behavioral effects of specific modification in the olfactory circuitry [11]. Therefore, we used this parameter as a readout to quantify and compare the behavior under head-restrained conditions towards different stimuli.

The correlation between task demands and reaction times have well been studied across different sensory modalities [23]. In the olfactory system of rodents, reaction times or discrimination times have been studied using different behavioral paradigms. Go/no-go task provided evidences for DTs being dependent on stimulus complexity [1], [11], where as read outs from a two-alternative choice reaction time paradigm supported either the dependence [2] or independence [3] of DTs on the stimulus complexity. As urgency influences reaction times [24], we measured DTs using go/no-go tasks in freely moving and head-restrained conditions, where reward is provided after a fixed period of stimulus presentation and removing incentives for a quick response. In both cases, DTs measured for simple and complex odorants provided evidence that DTs depend on the complexity of stimuli. Hence we show that in the olfactory system, like in other sensory systems, complexity influences the speed of neural processing. By obtaining similar behavioral readouts under head-restrained conditions and freely moving conditions, we show that the head-restrained strategy offers a robust and reliable method for dissecting the neural basis of olfactory specific behaviors.

## Materials and Methods

### Animals and Initial Preparation

All experiments were performed on male C57BL/6J mice (Charles River France) and were in accordance with the Swiss Federal Act on Animal Protection and Swiss Animal Protection Ordinance. The experiments were approved by the university of Geneva and Geneva state ethics committees (authorization 1007/3758/2).

Subjects were 10–12 weeks old at the beginning of behavioral experiments and were maintained on a 12 hr light-dark cycle in isolated cages in a temperature- and humidity-controlled animal facility. All behavioral training was conducted during daytime. During the training period, animals had free access to food but were on a water restriction schedule designed to keep them at >85% of their baseline body weight.

**Figure 1:**
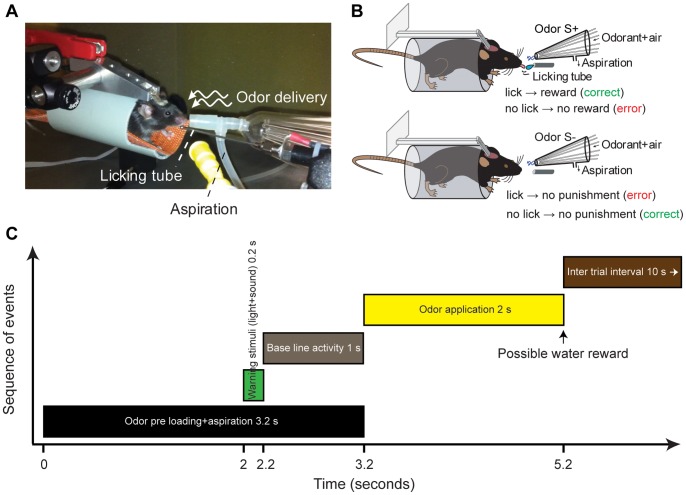
Go/no-go olfactory conditioning task under head-restrained conditions. (**A**) Mouse engaged in a go/no-go operant conditioning task. (**B**) Schema of rewarded and unrewarded trials. (**C**) Structure of a single trial. The criterion for a water reward was the total lick of 80 ms in three time bins of 500 ms out of four bins during the 2 s odor presentation, if there was no baseline licking. If mice were licking during the baseline, they had to lick double amount of time during the odor presentation to get the water reward.

For behavior experiments under head-restrained conditions, head post implantations were done as described previously (Fig. 1A, also see [25], [26]).

### Odorants

Odorants used were methyl benzoate (MB, ≥98% purity), amyl acetate (AA, ≥99%), ethyl butyrate (EB, ≥99%), Citral (Cit, >95%), 1-Butanol (But, ∼99%), cloves (clov) and camphor (cam). All chemicals and mineral oil were obtained from Sigma-Aldrich or Fluka Chemie (Steinheim, Germany).

#### Behavioral Training: Go/no-go task under head-restrained conditions

Before each behavioral session, a mouse was placed in a plastic tube and head-restrained by screwing the head post on a custom made metallic device fixed on a platform (Fig. 1A). All olfactory discrimination behavior experiments were done using a custom built olfactometer [27], [28] (for delivering the odorants), which was synchronized to a lickometer for recording the licking responses of mice towards different odorants (Fig. 1B).

Odor mixing and dilutions were achieved by the olfactometer through which a clean stream of air was split into a dilution stream and an odor stream that passed through bottles containing saturated odorants. These streams were merged right before the output at the level of an odor delivery nozzle (Fig. 1). Before odor presentation, odor flows were directed for 3.2 s into an exhaust circuit placed at the output to ensure that flows have reached a steady state (referred as preloading time in Fig. 1C), and were then sent to the nose by switching a valve. Computer driven mass flow controllers (Pneucleus Technologies LLC, Hollis, NH) and electromagnetic valves (SMC Pneumatik AG, Switzerland) allowed precise control of odor selection, dilution and timing. The steady state was assessed offline with a mass flow meter (Sierra Instruments Inc, Monterey, CA). The latter procedure was important to obtain a sharp stimulation onset and concentration stability during odor presentation. Further in the text, odor concentration is expressed as a percentage of the saturated vapor pressure, which reflects the relative flow rates of the odor and dilution streams. For example, a value of 5% means that the saturated odor stream was set at a flow rate 20 times lower than the dilution stream. In all cases, the total output flow rate was equal to 400 sccm (Standard Cubic Centimeter per Minute). Odorants were diluted to 1% in mineral oil (Fluka) and further diluted 1∶20 by airflow. Odors were freshly prepared for each task.

#### Task habituation training

Beginning 1–3 days after starting the water restriction schedule, animals were trained for an associative task using operant conditioning procedures (Fig. 1.B). In a first pre-training step, 3 s after the tone of 200 ms (two different frequencies, 5000 Hz and 6000 Hz, were used for two behavioral setups, as they were in the same room), a water drop (2 µl) was presented to animals irrespective of their responses (40 trials). This was meant to provoke the licking behavior of animals. During the second stage, following the tone, a baseline recording of licking (1 s) was performed before the odor presentation (in this experiment, 1% methyl benzoate). Usually odors were delivered by the olfactometers for 2 s. If animals were not licking during the baseline, we implemented the criteria for water delivery based on their licking time during the odor presentation. The total licking time required, during odor presentation, to trigger water reward was gradually increased in each step from 40 ms up to 240 ms (40 ms –30 trials, 80 ms –30 trials, 120 ms –30 trials, 160 ms –50 trials, 240 ms –50 trials). If animals were licking during the baseline, the required licking time kept increasing from 100% (same amount of licking as during the baseline) up to 200% (100% –30 trials, 125% –30 trials, 150% –30 trials, 175% –50 trials, 200% –50 trials). Essentially most animals learned this task in 2–3 days (4–6 sessions of 30 min each).

#### Olfactory behavioral training

Trial initiation was set by the experimenter with a constant inter-trial interval (13.2 s including the preloading of 3.2 s) between consecutive trials for all mice used in the experiment (Fig. 1C). The 3.2 s preloading of odorants and a very efficient aspiration system made the stimulus onset very precise and minimized the travelling time between “odor onset” and first contact of the animals’ nose with the odor. For an odor discrimination task, mice were usually trained for two odorants, one being rewarded (S+) and the other being unrewarded (S−). The required total licking duration for getting the water reward (2 µl) at the end of a rewarded trial was based on the licking activity during baseline. After the recording of baseline licking (if any) for 1 s, the odor was applied to the animal for 2 s. If mice licked during the baseline, they had to lick double amount of time during the odor presentation to get water reward. If mice were not licking during the baseline, the criterion for a water reward was a total lick time of 80 ms in three time bins of 500 ms out of four during the 2s odor presentation. Trials were counted as correct if the animal met with the abovementioned criteria for rewarded trials. For unrewarded trials, if mice were not licking during baseline, the criterion for a correct trial was a maximum lick time of 80 ms in one time bin of 500 ms out of four during the 2 s odor presentation. If mice licked during the baseline of an unrewarded trial, the trial was counted as correct if the total licking time during 2 s odor presentation did not exceed 25% of their baseline licking. Generally most of the mice did not lick for unrewarded trials and they consistently licked for rewarded trials after the task acquisition. No punishment was given to the mice for incorrect trials.

Odors were presented on a pseudo-randomized scheme (no more than 2 successive presentations of the same odor, equal numbers within each block of 20 trials, ensuring different order of presentations for S+ and S− trials within each 20 trial blocks). No intrinsic preference towards any of the odors was observed. Bias caused by odor preferences was generally avoided by counterbalancing S+ and S− stimuli such that each odor was designated S+ or S− stimulus for the same number of animals. A total of 200–300 trials per animal, separated into 30–40 min sessions to ensure maximal motivation despite the mildness of the water restriction scheme, were performed each day. Motivation was controlled by the frequency of licking. When the animals were unmotivated, they stopped responding to the rewarded trials. If the unmotivated trials were not observed by the experimenter during the training session, these were excluded from the final analysis.

At the initial periods of discrimination training (AA vs EB and their binary mixtures, see results) a warning signal of a tone and a green light pulse (200 ms) were delivered simultaneously 1 second prior to the 2 s odor presentations, in order to make the mice more alert. This was important as mice were required to respond to the trials delivered at inter-trial intervals set by the experimenter. As we did not observe any differences in the discrimination behaviors in the presence and absence of warning stimuli, the use of warning stimuli was avoided for further training sessions.

#### Measurement of discrimination times

The licking behavior of mice was monitored with a high temporal resolution of 2 ms. Upon presentation of a S+ odor, the animals continuously licked during the odor, whereas upon presentation of an S− odor mice hardly licked during the odor presentation. The average difference in response to the S+ and S− odor is approximately sigmoidal and yields a sensitive measure of the discrimination performance. Discrimination times were determined as follows: The blocks (20 trials) with ≥80% performance levels were selected and combined for 300 successive trials (150 S+ and 150 S−). For every 2 ms time point licking was monitored and then compared between S+ and S−, yielding significance value as a function of time after odor onset. The last crossing of the p = 0.05 line was measured by linear interpolation in the logarithmic plot and determined the discrimination time.

This DT analysis is optimized to identify the shortest reaction time occurring in the population of trials and is not affected by longer lasting events: Firstly, analyzing animals’ performance in blocks of 300 trials reduces the influence of variability such as potential variability in odor onset relative to the sniff cycle (first sniff cycle during odor presentation). Additionally, non-optimal sniff cycle onsets could result in a substantially delayed licking response for an S+ trial. Nevertheless, the time corresponding to first crossing of the p = 0.05 line (that is, the DT) will be delayed only by negligible amounts, provided that the number of optimal or near-optimal sniff cycle onsets is sufficiently large. Due to the fact that mice hardly licked for S− trials and because of reliable continuous licking upon presentation of S+ stimuli, (even they are few in number), resulted in significant difference between S+ and S− stimuli. This ensures further robustness against variability such as the potential variability of the sniff cycle relative to odor onset.

#### Behavioral Training: Go/no-go task under freely moving conditions

All discrimination experiments were performed using modified eight-channel olfactometers ([22]; Knosys, Washington) controlled by custom software written in Igor (Wavemetrics, OR). Behavioral training was executed as described elsewhere [1], [11]. In brief, odors were presented to the mice in a combined odor sampling/reward port. This ensured tight association of the water reward with the presented odorant. Head insertion into the port was monitored by an infrared beam and a photodiode. Odors were diluted to 1% in mineral oil (Fluka) and further diluted 1∶20 by airflow. Each rewarded (S+) and non-rewarded (S−) odor was presented from as many valves as possible (usually four each), allowing an online test of olfactometer integrity by comparing performance before and after switch of odor lines [e.g., Fig. 1C of [1]]. Odors were made freshly for each task.

One or two days prior to pretraining, mice were kept with restricted water access, and body weight was closely monitored. During pretraining sessions, animals were taught using standard operant conditioning procedures (See also [1]). In the first pre-training step, each lick at the water delivery tube was rewarded. After 20 licks a second stage began in which head insertion initiated a 2 s “odor” presentation during which a lick was rewarded. The “odorant” used in the pre-training was the mineral oil used for odor dilution. The complexity of the pretraining task increased gradually during five phases each consisting of 20 trials. Most animals learned this task within one day (2–3 sessions of 30 min each).

During olfactory training sessions, mice were trained to discriminate between two odorants, rewarded (S+) and unrewarded (S−). In brief, subjects initiated trials via infrared beam break at the sampling port opening. Then one of eight odor valves opened, as well as a diversion valve (DV) that allows all airflow to be diverted away from the animal for 0.5 s. After the release of DV, the odor was applied to the animal for 2 s. If the mouse continuously licked during this time (once in at least three out of four 500 ms bins), it received a 2 µl water reward after the end of the 2 s period. If the animal did not continuously lick, or if the presented odor was an S− odor, there was no reward. Odors were presented in a pseudorandomized scheme (no more than two successive presentations of the same odor). Upon presentation of a S+ odor, the animal generally continuously breaks the beam and licks on the water delivery tube, whereas upon presentation of an S− odor an animal familiar with the apparatus usually quickly retracts its head. DTs were calculated as follows: for every time point, beam breakings for S+ and S− odors were compared by bootstrapping, yielding significance value as a function of time after odor onset. The last crossing of the p = 0.05 line determined the DT. In very few cases, this did not coincide with the visually identified discrimination time [point of largest curvature in the log(p)-t plot] and was corrected after visual inspection.

## Results

### Head-restrained Mice can Learn to Accurately Discriminate both Simple and Complex Odorants

**Figure 2:**
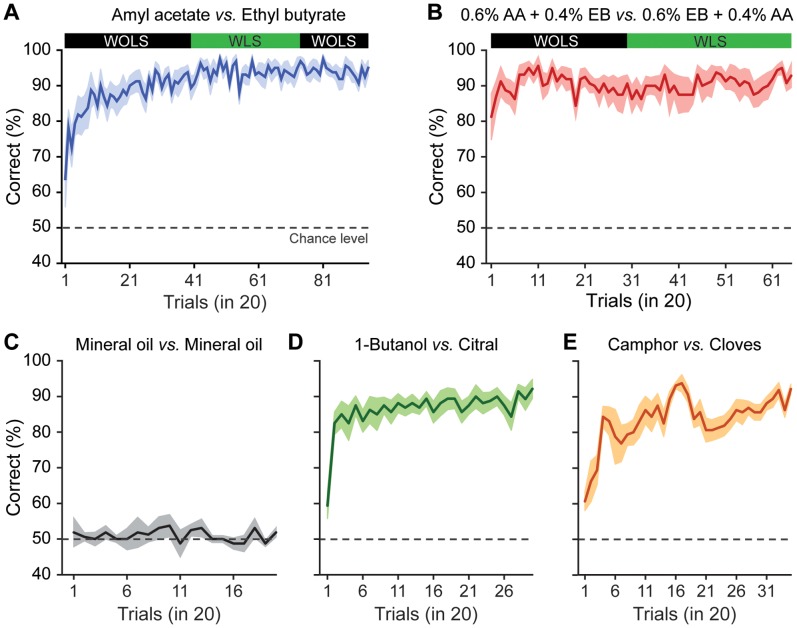
Mice learn simple as well as complex odorants with high accuracy under head-restrained conditions. Discrimination accuracy shown as the average percentage of correct choices: (**A**) amyl acetate vs ethyl butyrate (n = 8 mice, average ± sem), (**B**) complex binary mixtures of AA and EB, 0.6% AA +0.4% EB vs. 0.6% EB +0.4% AA (n = 8 mice, average ± sem). (**C**) Learning observed in all cases is olfactory specific, mice showed no learning when they were trained on a mineral oil vs mineral oil task (n = 8 mice, average ± sem). (**D**) citral vs 1-butanol (n = 8 mice, average ± sem) (**E**) cloves vs camphor (n = 8 mice, average ± sem).

Olfactory discrimination was investigated by training mice on a go/no-go operant conditioning paradigm under head-restrained conditions (Fig. 1A–C, see materials and methods). Their olfactory responses were evaluated by licking behavior in response to odorants with a temporal precision of 2 milliseconds. After acquiring the procedural aspects of olfactory tasks, mice were trained to discriminate many different odor pairs including complex binary mixtures and natural odorants. Mice rapidly learned to discriminate amyl acetate (AA) vs ethyl butyrate (EB) and their complex binary mixtures (0.6% AA +0.4% EB vs 0.6% EB +0.4% AA) with high accuracy (Fig. 2A, 2B). While the mice were acquiring these discrimination tasks, a warning signal of a tone and a green light pulse (200 ms) were delivered simultaneously 1 second prior to the 2 second application of odor stimulus, in order to alert the mice about incoming olfactory stimulus (see materials and methods). This was important as we were setting the inter-trial intervals (based on the results from freely moving mice) for the initiation of trials under head-restrained conditions. In order to investigate the effect of using non-olfactory cues prior to olfactory cues, their performance levels were compared and found no difference for simple as well as complex discrimination tasks [Fig. 2A, 2B, AA/EB simple discrimination task, comparison between 400 trials of second and third blocks: ANOVA, p = 0.93 (first block was avoided as this was involving the learning of odorants as well, AA/EB binary mixture discrimination task, comparison between 600 trials of first and second blocks: ANOVA, p = 0.96], discarding a possible effect from the use of warning stimuli. Since the animals were learning the odor pairs even in the absence of warning stimuli, no warning stimulus was used for the discrimination tasks of mineral oil vs mineral oil (MO), Citral (Cit) vs 1-Butanol (But) and Cloves (Clov) vs Camphor (Cam). As we observed efficient learning for AA/EB and their binary mixtures, we trained the mice for a MO vs MO (solvent used to dilute all odorants) task and found no significant learning even after 400 trials, indicating that mice were only learning the olfactory cues (Fig. 2C, ANOVA p = 0.97, N = 8 mice). When the same mice were trained for another odor pair, Cit vs But, they reached a performance level of higher than 80% in tens of trials (Fig. 2D). Finally the mice were trained on a natural odorant discrimination task, Clov vs Cam, which they acquired rapidly with high accuracy (Fig. 2E). In conclusion, mice can learn simple as well as complex olfactory discrimination tasks rapidly with high accuracies under head-restrained conditions.

### Rapid and Stimulus Dependent Discrimination Times are Observed in a Go/no-go Olfactory Task Under Head-restrained Conditions

**Figure 3:**
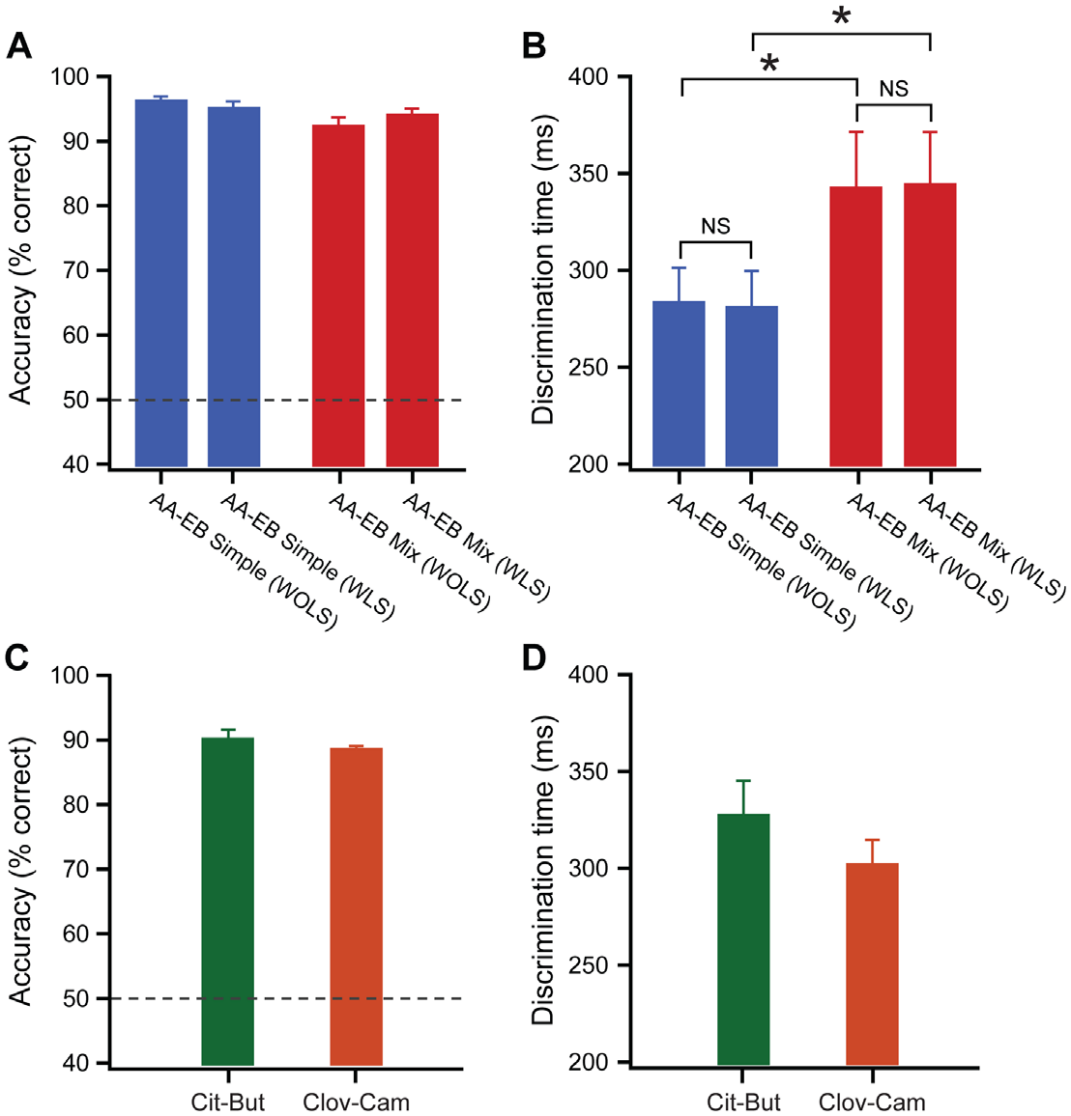
Rapid and stimulus dependent discrimination times are observed in a go/no-go olfactory task under head-restrained conditions. (**A–B**) Determination of discrimination times in presence (WLS) and absence of warning stimuli (WOLS): No influence of warning stimuli (light+sound) in the DT measurements. (WOLS = Without Light and Sound, WLS = With Light and Sound). (**A**) Accuracies measured for simple and complex odor tasks of amyl acetate and ethyl butyrate, 300 trial blocks from which DT is calculated (for AA vs EB, blue bars, n = 8 mice, average ± sem; for binary mixtures, red bars, n = 8 mice, average ± sem). (**B**) DTs were increased for complex odors compared to simple odors even in the presence and absence of warning stimuli (for AA vs EB, blue bars, n = 8 mice, average ± sem; for binary mixtures, red bars, n = 8 mice, average ± sem. (**C–D**) Accuracy and discrimination times measured for cit vs but and clov vs cam, 300 trial blocks from which DT is calculated (for cit vs but, green bar and clov vs cam, brown bar, n = 8 mice, average ± sem). Dotted lines in (A) and (C) indicate chance levels.

As mice performed accurately for all odor pairs tested we tried to look for a more sensitive readout, the time needed for such accurate discriminations (discrimination time; DT), a well established parameter to monitor olfactory behavior in freely moving mice [1], [2], [3], [11]. We took advantage of the responses towards rewarded trials as mice consistently licked for these trials when they were performing accurately. To measure DTs, licking responses towards the rewarded and unrewarded odors were monitored with a temporal precision of 2 ms and compared to each other. In a freely moving go/no-go operant conditioning task, mice decide when to initiate trials, whereas under head-restrained conditions we set the inter-trial interval (based on the average inter-trial interval observed in all behavioral experiments done in different laboratories) to start the trials. This resulted in random unmotivated trial blocks with no behavioral responses even after mice learned the task. To avoid the possible bias from these unmotivated trials, we selected blocks (20 trials) with ≥80% performance levels for calculating the DTs. For comparison of DTs, the number of trials from simple and complex odor tasks was kept the same for individual mice and the performance levels at these blocks of trials were always high (Fig. 3A). Mice discriminated simple odor pairs of AA/EB quickly, while for complex binary mixtures of AA/EB (0.6% AA +0.4% EB vs. 0.6% EB +0.4% AA) they took tens of milliseconds longer (Fig. 3B), confirming the stimulus dependency of olfactory DTs.

Since we used the warning stimulus while mice learned these tasks, DTs were also compared for the presence and absence of this warning stimulus. One possible effect of this non-olfactory cue is that the mice may use these cues alone or along with olfactory stimuli to initiate licking. When the DT’s were measured from the highest performing blocks of simple and complex odor pairs (Fig. 3A, See also Figs. 2A and 2B for the complete sequence of training), we found no difference in the DT measurements in presence and absence of warning stimulus (Fig. 3B), discarding a possible bias from the use of such stimuli and thereby confirming the learning of olfactory stimuli during the task. Since we observed the same DTs under both conditions, for the following tasks of Cit vs But and Clov vs Cam, no warning stimulus was used.

Followed by the MO vs MO task which confirmed olfactory specific learning, mice were further trained for other odor pairs Cit vs But and Clov vs Cam, during which they performed well and DTs were measured from these high accuracy blocks (Fig. 3C). Mice discriminated these odor pairs quickly thereby accumulating the evidence for fast sensory processing in rodent olfaction (Fig. 3D). In summary, DTs measured in a go/no-go task under head-restrained conditions was fast and critically depended on stimulus where the measurements were primarily based on responses towards rewarded odorants.

### Head-restrained Mice Show Similar Discrimination Abilities as Freely Moving Mice

Mice showed robust discrimination abilities over months when they were trained for different simple and complex odor pairs under head-restrained conditions. As this method is useful for monitoring neuronal activity while the animals are actively involved in a behavioral assay, we next investigated if the discrimination abilities are comparable with freely behaving animals. To study this question, different batches of wild type mice were trained on a go/no-go operant conditioning task [1], [22] for the same odor pairs, and behavior readouts were compared.

**Figure 4:**
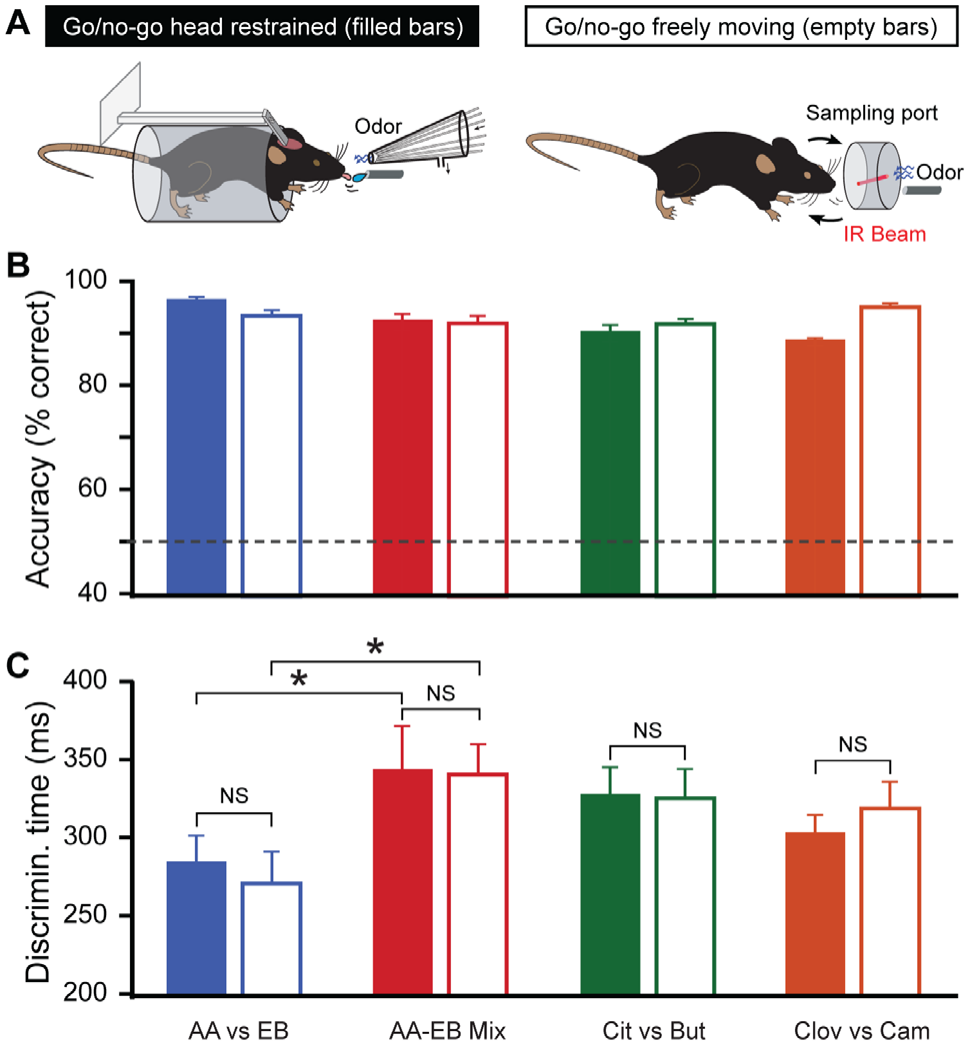
Similar discrimination abilities were observed under freely moving and head-restrained conditions. (**A**) Schema depicting head-restrained and freely moving paradigms. (**B**) Discrimination accuracy shown as the average percentage of correct choices, 300 trial blocks from which DT is calculated (filled bars represent head-restrained conditions on a go/no-go task and empty bars represent freely moving conditions on a go/no-go task) for AA vs EB (blue, n = 8 mice, average ± sem), binary mixtures of AA and EB (red, n = 8 mice, average ± sem), cit vs but (green, n = 8 mice, average ± sem) and clove vs cam (brown, n = 8 mice, average ± sem). Dotted line indicates chance level. (**C**) Stimulus dependent DTs were observed for go/no-go conditioning task under freely moving conditions and were similar to the DTs measured under head-restrained conditions (color codes and number of mice: same as above, all values are expressed as average ± sem).

After the task habituation training, when naïve mice were trained on a go/no-go operant conditioning paradigm (Fig. 4A) for different odor pairs including AA/EB, AA-EB binary mixtures, Cit/But and Clov/Cam, they acquired the discrimination in few hundreds of trials (Fig. S1) thus stabilizing their performance levels at more than 80% (Fig. 4B, Fig. S1). After observing high performance levels for these odor pairs, we next measured the discrimination times, a more sensitive readout [11] for evaluating the olfactory behavior. In the case of the go/no-go operant task under head-restrained conditions, DTs are more influenced by the response behavior (licking) towards a positively rewarded odor where as in the go/no-go task under freely moving conditions, DTs are determined by the head withdrawal behavior shown by mice towards the unrewarded trials (Fig. 4A). DTs were measured while the animals were performing well above 80% (Fig. 4B) as described in [1]. When we compared the DTs measured for simple as well as complex odors, head-restrained mice showed similar DTs compared to the freely moving mice (Fig. 4C), showing once again that the head-restrained protocol did not affect the discrimination abilities of mice.

In conclusion, the head-restrained go/no-go operant conditioning paradigm resulted in similar behavioral readouts to those we observed with the freely moving animals, showing the reliability of this method in evaluating olfactory behavior with high temporal precision.

## Discussion

Here we show that mice can learn both simple and complex odorants rapidly with high accuracy (Figs. 2 and 3) under head-restrained conditions. In addition, the behavioral readouts we obtained under head-restrained conditions were similar to those obtained from freely moving mice (Fig. 4). Discrimination time measurements were based on responses monitored by lick detection measured at 2 ms temporal resolution. Strikingly the rapid and stimulus dependent discrimination times occurred even when the measurements were based on responses towards positively rewarded olfactory stimuli and were comparable to the time measurements that were primarily based on the responses towards unrewarded stimuli in freely moving animals. These results thereby provide evidence for the commonality of olfactory system with other sensory systems in temporal processing of sensory signals.

### Stable Olfactory Discrimination Performances over Long Time Periods

One of the major challenges in understanding how the brain functions is real time correlation of neuronal activity at the single cell and network level with behavioral outputs over long time periods. Rodents can learn to discriminate simple and complex odor mixtures upon training [1], [2], [29]. As the primary relay centre of olfactory information processing, the olfactory bulb of rodents is easily accessible for *in vivo* monitoring of neuronal activity using electrophysiological and imaging approaches; studying olfactory discrimination behavior, therefore, offers an attractive model for exploring the neural basis of behavior. The difficulty of monitoring physiological parameters such as spikes or calcium activity in freely moving animals [30], [31] makes head-restrained strategy more efficient [19], [26], [32], [33], [34], [35]. To our knowledge, a study that compares different behavioral outputs under head-restrained and freely moving conditions is still missing. In this report we did a systematic comparison of different behavioral readouts from these two conditions. After the acquisition of procedural aspects of training through a pre-training session and a simple discrimination task, head-restrained mice learned quickly to discriminate novel odor pairs, reaching 80% accuracy in few tens of trials (Fig. 2), compared to go/no-go freely moving paradigm (Fig. S1, cf. Figs. 1B, 4A1 of [1], and Figs. 5A, 7A of [11]). Although the exact reason for this still needs to be investigated, one simple explanation is the extra motivation brought by the enforcement of activity under head-restrained conditions whereas in freely moving conditions, animals are free to act according to their choice. More notably, the accurate performance for the simple and complex discriminations were continued for hundreds of trials over many days (1900 trials for AA-EB simple task and 1300 trials for AA-EB mixture task, average number of trials performed in a single day being 200). This stability of performance levels makes this approach suitable for long term monitoring of neural activities while the animals are actively involved in olfactory behavior.

### Stimulus Dependant Discrimination Times

Stimulus-dependency of olfactory discrimination times remains an intensively discussed topic [1], [2], [3], [4], [36], [37], [38], [39]. Our previous results, along with others, have provided evidence that complex odorants require longer DTs than simple monomolecular odorants in mice [1], [2]. In these studies, mice were able to maintain accurate performance for complex discriminations by taking additional milliseconds for decisions. But olfactory discrimination studies done in rats support the notion of stimulus independent DTs, although the accuracy drops for complex discriminations [3]. In go/no-go freely moving behavioral paradigms, DT measurements are primarily based on the head withdrawal behavior towards unrewarded trials where as in go/no-go tasks under head-restrained conditions the DT measurements are resulted from animal’s licking behavior, a reaction mainly towards positively rewarded stimuli at highly learned stages. Therefore, we were able to measure and compare the DTs using two different strategies. Our results here provide evidence for the stimulus dependent nature of discrimination times, consistent with the reaction time measurements from other sensory systems [23].

### Advantages of Studying Olfactory Behavior Under Head-restrained Conditions

One important requirement for recording neuronal activities in awake animals is stability. Although chronic recording of OB activity has been done [30], [31], these attempts have been made in rats. The small size of OB makes these experiments challenging in mice, the model system that offers the possibility for specific genetic manipulations in the neuronal circuitry. Therefore, the best strategy for correlating neuronal activity with behavior is to perform the experiments under head-restrained conditions. One of the behavioral readouts from our experiments, DT measurements, provides the time window for the temporal integration of olfactory information and decision making processes. In this highly reliable behavioral paradigm, we were able to record the licking responses to calculate DTs at a time resolution of 2 ms. With the emerging optogenetic tools and genetically-encoded activity indicators to monitor neuronal activities over long time periods [13], [40], [41], [42], [43], [44], this behavioral paradigm will help to study the neural basis of specific olfactory driven behaviors and decision making processes.

## Supporting Information

Figure S1 **Mice learn simple as well as complex odorants with high accuracy under freely moving conditions.** Discrimination accuracy shown as the average percentage of correct choices for (**A**) amyl acetate vs ethyl butyrate (n = 8 mice, average ± sem), (**B**) complex binary mixtures of AA and EB, 0.6% AA +0.4% EB vs. 0.6% EB +0.4% AA (n = 8 mice, average ± sem), (**C**) citral vs 1-butanol (n = 8 mice, average ± sem) and (**D**) cloves vs camphor (n = 8 mice, average ± sem, performance started very high because the same mice were trained for another similar discrimination task [1,4-cineol vs eugenol] immediately before cloves vs camphor task).
(TIF)
