# Supplementary figures and images for: Similar Odor Discrimination Behavior in Head-Restrained and Freely Moving Mice
Source: PLoS One. 2012 Dec 18;7(12):e51789. doi: 10.1371/journal.pone.0051789 (PMC3525655; doi:10.1371/journal.pone.0051789)

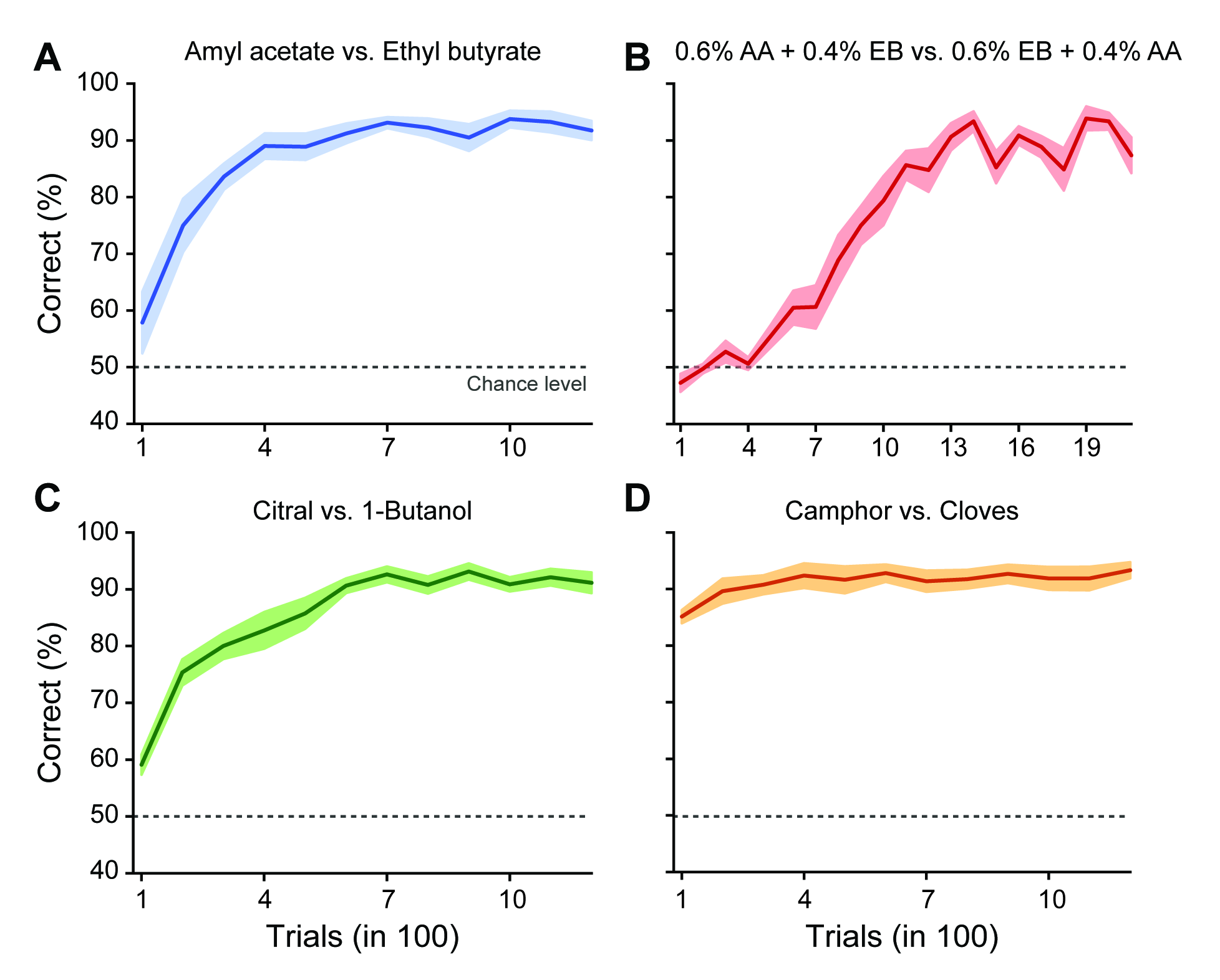

Supplement: Figure S1 — Mice learn simple as well as complex odorants with high accuracy under freely moving conditions. Discrimination accuracy shown as the average percentage of correct choices for (A) amyl acetate vs ethyl butyrate (n = 8 mice, average ± sem), (B) complex binary mixtures of AA and EB, 0.6% AA +0.4% EB vs. 0.6% EB +0.4% AA (n = 8 mice, average ± sem), (C) citral vs 1-butanol (n = 8 mice, average ± sem) and (D) cloves vs camphor (n = 8 mice, average ± sem, performance started very high because the same mice were trained for another similar discrimination task [1,4-cineol vs eugenol] immediately before cloves vs camphor task). (TIF) [file pone.0051789.s001.tif]
